# Resection of piriform cortex predicts seizure freedom in temporal lobe epilepsy

**DOI:** 10.1002/acn3.51263

**Published:** 2020-12-02

**Authors:** Valeri Borger, Matthias Schneider, Julia Taube, Anna‐Laura Potthoff, Vera C. Keil, Motaz Hamed, Gülsah Aydin, Inja Ilic, László Solymosi, Christian E. Elger, Erdem Güresir, Rolf Fimmers, Patrick Schuss, Christoph Helmstaedter, Rainer Surges, Hartmut Vatter

**Affiliations:** ^1^ Department of Neurosurgery University Hospital Bonn Bonn Germany; ^2^ Department of Epileptology University Hospital Bonn Bonn Germany; ^3^ Department of Neuroradiology University Hospital Bonn Bonn Germany; ^4^ Institute of Medical Biometry Informatics and Epidemiology University Hospital Bonn Bonn Germany

## Abstract

**Objective:**

Transsylvian selective amygdalo‐hippocampectomy (tsSAHE) represents a generally recognized surgical procedure for drug‐resistant mesial temporal lobe epilepsy (mTLE). Although postoperative seizure freedom can be achieved in about 70% of tsSAHE, there is a considerable amount of patients with persisting postoperative seizures. This might partly be explained by differing extents of resection of various tsSAHE target volumes. In this study we analyzed the resected proportions of hippocampus, amygdala as well as piriform cortex in regard of postoperative seizure outcome.

**Methods:**

Between 2012 and 2017, 82 of 103 patients with mTLE who underwent tsSAHE at the authors’ institution were included in the analysis. Resected proportions of hippocampus, amygdala and temporal piriform cortex as target structures of tsSAHE were volumetrically assessed and stratified according to favorable (International League Against Epilepsy (ILAE) class 1) and unfavorable (ILAE class 2–6) seizure outcome.

**Results:**

Patients with favorable seizure outcome revealed a significantly larger proportion of resected temporal piriform cortex volumes compared to patients with unfavorable seizure outcome (median resected proportional volumes were 51% (IQR 42–61) versus (vs.) 13 (IQR 11–18), *P* = 0.0001). Resected proportions of hippocampus and amygdala did not significantly differ for these groups (hippocampus: 81% (IQR 73–88) vs. 80% (IQR 74–92) (*P* = 0.7); amygdala: 100% (IQR 100–100) vs. 100% (IQR 100–100) (*P* = 0.7)).

**Interpretation:**

These results strongly suggest temporal piriform cortex to constitute a key target resection volume to achieve seizure freedom following tsSAHE.

## Introduction

Temporal lobe epilepsy (TLE) is one of the most common entities of epilepsy, first described by Hughlings‐Jackson in 1898.^1^ In approximately 30% of patients, seizures are refractory to drug treatment.^2^, ^3^ Since the first randomized controlled trial by Wiebe et al. has shown significantly improved outcomes with epilepsy surgery over drug treatment in refractory TLE, resective temporal lobe surgery (rTLS) has become a reasonable option for treatment in these patients.^4^ Especially for the treatment of mesial TLE (mTLE), a selective amygdalo‐hippocampectomy via the transsylvian approach (tsSAHE) was introduced.^5^ The aim of this approach was to perform a lesionectomy of mesiotemporal structures avoiding trauma to the adjacent healthy temporal neopallial areas and to the vasculature.^6^ Despite reported seizure freedom rates between 60% and 70%, there is a considerable amount of patients with continued seizures after epilepsy surgery.^7^ The reasons behind failure of surgery for mTLE are diverse and vary between cases. In the previous decades, many efforts were made to identify a sufficient resection extent of mesiotemporal structures.^8^, ^9^, ^10^, ^11^, ^12^ However, results from these studies have been conflicting and the issue of optimal extent of resection remains controversial.^13^

A recently published study by Galovic et al. reported strong evidence for the association of piriform cortex resection with surgical seizure outcome in patients with TLE who underwent a standard anterior temporal lobe (ATL) resection.^14^ Thus, extended removal of the piriform cortex has been shown to significantly increase the probability of becoming seizure free. Against this backdrop, the piriform cortex seems to constitute a novel key target volume for ATL resections. However, the impact of piriform cortex resection in the setting of transsylvian SAHE for treatment of mTLE is still unknown. Therefore, the aim of this study was to investigate whether the extent of piriform cortex resection might significantly contribute to postoperative seizure outcome in the course of tsSAHE.

## Methods

### Patient population and presurgical evaluation

Patients with TLE who underwent rTLS between 2012 and 2017 were reviewed from the prospectively kept epilepsy surgery database at our hospital. The establishment of this database was approved by the local ethics committee. Informed consent was not sought as a retrospective design was used.

During the studied period, rTLS was performed in a total of 184 patients. For patients suffering from unilateral mTLE, in our center the tsSAHE is the first‐line surgical treatment option. The rationale for surgical procedure selection was based on the magnetic resonance imaging (MRI)‐documented pathological lesion as well as putative epileptogenic tissue volumes as suggested in the course of preoperative clinical and electroencephalographical evaluation as previously described for our interdisciplinary epilepsy center.^15^

All patients were presurgically assessed in the department of epileptology and were considered to be suitable for surgery.^16^, ^17^, ^18^ The evaluation included detailed history of seizures, medical history, high resolution structural 3.0 Tesla MRI, neuropsychological assessment,^19^ and video‐electroencephalography (EEG) monitoring using continuous recordings. In patients with absent or several lesions on MRI – the latter defined as any coexistent extratemporal lesion beyond the ipsilateral temporal lobe that had undergone surgery for tsSAHE – positron emission tomography (PET) and single‐photon emission computed tomography (SPECT) were performed in order to identify a seizure focus. In cases with nonconclusive findings, invasive EEG monitoring was performed using stereotactically implanted depth electrodes.^20^ Following completed evaluation, the extent of resection was determined in every individual candidate by the interdisciplinary epilepsy conference.

Accordingly, the tsSAHE was performed in 103 consecutive patients. To establish a uniform code of study quality, we only included patients with (a) at least completed 12 months of follow‐up after surgery; (b) available pre‐ and postsurgical structural magnetic resonance imaging (MRI) scans acquired according to the identical scanning protocol and (c) tsSAHE that was performed as a highly standardized surgical procedure by three neurosurgeons (H.V., V.B., M.H.). Considering the abovementioned inclusion criteria, 12 patients were lost to follow‐up and nine patients had limited postoperative MRI‐protocols. A total of 82 eligible patients were included in the analysis. All patients suffered from medically refractory mTLE and had undergone adequate treatment with at least two first‐line antiepileptic drugs (AED).

### Surgical procedure of transsylvian selective amygdalo‐hippocampectomy

All surgical procedures were performed under general anesthesia using intraoperative neuronavigation and intraoperative neurophysiological monitoring with motor evoked potentials (MEP) and somatosensory evoked potentials (SSEP). The goal of surgery was to anatomically remove mesiotemporal target structures that entail presumed seizure focus. For SAHE, exclusively the transsylvian approach as described by Yasargil et al.^6^ with several modifications was used by all neurosurgeons. The surgery was performed as a highly standardized procedure. Shortly described, the patient is positioned supine, the head is fixed in Mayfield‐Clamp turned 45° to the opposite side, and the vertex is tilled 5–10° to the bottom. A standard pterional craniotomy was performed. Attention was paid to extend the frontal margin of the bone flap to the mediopupillar line. After opening of the dura, the proximal sylvian fissure was dissected and the sylvian fossa was exposed. In the next step, the deep sylvian vein and limen insulae were identified and a small pial incision into the piriform cortex, 2–3 mm lateral to the M_1_ segment and lateral to the deep sylvian vein, was performed. The resection of the superior and lateral parts of the amygdala was performed using neuronavigation and ultrasound suction system (CUSA) just along the lateral border and ventral to the tip of the temporal horn until the temporobasis was reached. From this point, the further resection of amygdala and uncus was performed using CUSA or Penfield dissector, until the pial and arachnoid membranes, adjacent to the crural and ambient cisterns, was reached. The temporal horn was then opened using a dissector and the coroidal point was identified followed by resection of the anterior part of the hippocampus. The body of the hippocampus was then resected en bloc and obtained for histopathological analysis. The resection of dorsal parts of the hippocampus was extended until the level of the tectum.

### Imaging and volumetric analysis

All MRI studies were performed pre‐ and postoperatively at the same 3.0 Tesla scanner (Achieva TX, Philips Healthcare, Best, the Netherlands) with identical scanning protocols. All patients underwent MRI within 2–3 days postoperatively in order to detect the extent of resection of desired structures. The pre‐ and postoperative scans were measured as a pair by two independent and blinded raters. For postoperative assessment the same landmarks were used and preoperative outlines were transposed onto postoperative scans. The volumetric analysis was performed by A.‐L.P. and M.S. after training and under continuous supervision provided by V.C.K. and L.S. (8 and 25 years of experience in tumor volumetry) using commercially available software (Intellispace 8.0, Philips Healthcare, Best, the Netherlands). V.C.K. checked and analyzed data for accuracy and methodological consistency afterwards. The volumes of amygdala, hippocampus and piriform cortex were obtained from presurgical and postsurgical 1mm isovoxel 3D T1‐weighted MPRAGE scans. The resected proportions of amygdala, hippocampus and piriform cortex were then calculated in each individual. In this series, the segmentation and volumetry of hippocampus, amygdala and piriform cortex were manually performed by trained raters under continuous supervision by experienced neuroradiologists. Each hippocampus was traced in coronal, axial and sagittal plane. The entire length of each hippocampus was manually outlined to anatomic landmarks. The anterior border of the hippocampus was defined by distinguishing from amygdala by the presence of the alveus or the cerebrospinal fluid (CSF) of the lateral ventricle (e.g. uncal recess).^21^, ^22^ The posterior border was reached when the fornices were visible in their full length in sagittal plane. The anterior mesial border was defined by the posterior portions of the uncal fissure whereas the posterior mesial border was made up by the open end of the hippocampal fissure. The CSF of the lateral ventricle defines the lateral boundary of the hippocampus. The white matter of the parahippocampal gyrus below the subiculum defines the inferior limit. The manual volumetrical segmentation of the amygdala was performed according to existing protocols.^23^ In short, the posterior border was defined in the coronal plane by the alveus that appears inferiorly to the amygdala and the head of the hippocampus, which is inferior‐medial. The axial plane was used to identify the medial and lateral border. The ambient cistern limited the medial boundary. The lateral border was defined by the inferior horn of the lateral ventricle. For the identification of the inferior border, the amygdala was traced in the coronal slices. The tentorial indentation was a demarcation line between amygdala and entorhinal cortex. The anterior limit was defined at the level of the closure of lateral sulcus, which could easily be found in the axial sections.

For volumetric analysis of piriform cortex, we basically employed the work reported by Vaughan and Jackson. Thus, the human piriform cortex was subdivided in a frontal and a temporal part. In the temporal lobe, the piriform cortex becomes contiguous to periamygdaloid cortex both anatomically and functionally, and posteriorly extends to overlie the amygdala complex. Medially, piriform cortex limits to the entorhinal cortex with the sulcus semiannularis as its border. In the frontal lobe, the piriform cortex extends from the fundus of the entorhinal sulcus, and is limited medially by the olfactory tubercle and the lateral olfactory tract. The extent of the piriform cortex in the posterior – anterior direction in coronal plane begins at the level of the opening of the hippocampal fissure. From this level, the anterior limit is at the level of the limen insulae. The frontal part of the piriform cortex was definded in accordance to Vaughan and Jackson^24^ as a triangular region, which starts from the fundus of the endorhinal sulcus and is bounded medially by the olfactory tubercle and the lateral olfactory tract. The boundary between the superior medial border of the amygdala and piriform cortex is represented by the periamygdaloid cortex (PAC) area. Given by the fact, that in the MR imaging, the discrimination between PAC and piriform cortex is not possible, we grouped the piriform cortex and PAC together for further volumetric analysis. This approach is feasible because both these areas are closely connected both spatially and functionally and were also previously reported by Conçalves Pereira et al.^25^

Due to its eloquent localization and inherent risk of vascular damage, the frontal part of the piriform cortex was not intended for resection during tsSAHE. Given by this fact, we consequently excluded the frontal part of the piriform cortex for volumetric analysis.

### Seizure outcome analysis

Seizure outcome was assessed during follow‐up visits at 6 and 12 months. At the 12 months visit, all patients underwent thorough clinical examination, evaluation of seizure outcome, Video‐EEG recording, high resolution structural 3 Tesla MRI, and neuropsychological reassessment. Postoperative seizure outcome was assessed according to the ILAE classification.^26^ Patients were divided into two groups according to the seizure outcome (group I: ILAE class 1; group II: ILAE class ≥ 2). The ILAE class 1 outcome was considered favorable, the ILAE class ≥ 2 outcome unfavorable.

### Statistical analysis

Statistical data analysis was performed using software package SPSS (IBM SPSS Statistics for Windows, Version 25.0. Armonk, NY: IBM Corp.). Associations between parametric variables were analyzed using unpaired, two‐tailed Student t‐test. The Mann‐Whittney‐U test was chosen to compare continuous variables as the data were largely not normally distributed. Associations of categorical variables were compared using Chi‐square test or Fisher exact test. Results with *P* < 0.05 were considered to be statistically significant. For identification of independent risk factors for unfavorable postoperative seizure outcome (ILAE class ≥ 2), a two‐level logistic regression analysis was performed including the variables with significant *P*‐values in univariate analysis. The results of the analysis were presented as odds ratios (OR) with 95% confidential interval (CI). Finally, receiver operating characteristic (ROC) analysis was performed to explore the power of the resulting model.

### Neuropsychological assessment

The neuropsychological evaluation focused on tests of verbal and nonverbal memory representing temporal lobe functions. In addition, attention and executive functions, visuo‐spatial abilities, language and motor functions were considered. Verbal learning and memory were measured via the VLMT, a German adaptation of the Rey Auditory Verbal Learning Test.^27^ For nonverbal learning and memory the revised version of the DCS‐R was applied.^28^ Parallel versions of the VLMT and DCS‐R were employed to minimize practice effects at the follow‐up. Attention was assessed by the EpiTrack^29^ and a letter cancellation task. Language assessment comprised confrontation naming and a comprehension task (Token Test). Evaluation of visuo‐spatial abilities was carried out by administering mental rotation and WAIS block design. The tests and their references are described in previous articles.^30^

Pre‐ and postoperative test results from each cognitive domain were summarized and classified into a five‐point scale ranging from severely impaired to above average (severely impaired = 0, at least two test scores > 2SD below the mean of the normative sample; impaired = 1, at least two test scores > 1SD below the mean; borderline = 2, one test score below the mean; unimpaired = 3, no test score > 1SD below the mean; above average = 4, at least two test scores > 1SD above the mean). The distance between two subsequent categories approximately corresponds to one SD from the mean standardized score across all test scores of the respective domain.^31^

### Statistical analysis

The neuropsychological outcome was defined as the intraindividual change of cognitive categories from pre‐ to postoperative assessment. Therefore, we subtracted the postoperative from the preoperative category score per domain. A positive value indicated improvement, a negative value indicated deterioration, a value of zero indicated no change.^30^ To investigate postoperative changes depending on the side of surgery we performed chi‐square tests for each cognitive domain (attention, verbal memory, visual memory, language). In addition, language was examined with separate repeated measures analyses of variance with raw scores from the Token Test, Boston Naming Test and phonemic fluency as within‐subjects factors and side of surgery as the between‐subjects factor. We chose nonparametric tests, since our data were not normally distributed. To predict which factors influence the postoperative cognitive outcome, separate multiple linear regressions (enter method) were calculated for each cognitive domain. As potential predictors we included the extent of resection of piriform cortex, baseline performance, seizure freedom, and side of surgery as independent variables.

## Results

### Patient characteristics

Between 2012 and 2017, 82 patients with drug‐resistant mTLE underwent tsSAHE at the authors’ institution. Postoperative seizure freedom in terms of ILAE class 1 could be achieved for 59 patients (72%) (Table 1).

**Table 1:** Baseline patient characteristics according to ILAE class seizure outcome^1^.

Characteristics	ILAE class 1 (*n* = 59)	ILAE class 2–6 (*n* = 23)	*P* Value
Sex			
Male, *n* (%)	28 (47)	14 (61)	0.33
Female, *n* (%)	31 (53)	9 (39)	
Age at epilepsy onset (mean years ± SD)	16.8 ± 14.2	17.0 ± 12.1	
Duration of epilepsy (mean years ± SD)	22.7 ± 13.2	20.0 ± 16.4	
Age at surgery (mean years ± SD)	39.2 ± 14.1	36.2 ± 14.2	
Site of surgery			
Left, *n* (%)	30 (51)	12 (52)	1.0
Right, *n* (%)	29 (49)	11 (48)	
Invasive presurgical evaluation with depth electrodes *n* (%)	12 (20.3)	9 (39)	0.1
Preoperative MRI findings			
Unilateral hippocampal sclerosis, *n* (%)	52 (88.1)	16 (69.6)	0.06
No lesion, *n* (%)	4 (6.8)	5 (21.7)	0.11
Hippocampal gliosis, *n* (%)	1 (1.7)	2 (8.6)	0.19
Unspecific hippocampal lesion	2 (3)	0 (0)	1.0
Coexistent lesions^2^	10 (17)	4 (21)	1.0
Histology of hippocampus			
Hippocampal sclerosis, *n* (%)	52 (88.1)	15 (65.2)	0.03
Hippocampal gliosis, *n* (%)	7 (11.9)	6 (26.1)	0.18
Others, *n* (%)	0 (0.0)	2 (8.7)	0.08
Peri‐ and postoperative complications	2 (3)	3 (13)	0.1

ILAE, International League Against Epilepsy; SD, standard deviation.

^1^ Values represent number of patients unless otherwise indicated (%).

^2^ Defined as any coexistent extratermporal lesion beyond the ipsilateral temporal lobe of surgery.

Side of surgery did not significantly impact the postoperative seizure outcome: 30/42 patients (71%) were seizure free after left tsSAHE and 29/40 patients (73%) were seizure free after right tsSAHE (*P* = 1.0).

In the course of pre‐surgical evaluation, invasive diagnostics using depth electrodes were performed in 12 patients (20%) within the ILAE class 1 group and nine patients (39%) within the ILAE class 2–6 group (*P* = 0.1). The histopathological analysis revealed that 52 out of 67 (88%) patients with hippocampal sclerosis had a favorable outcome, compared to seven out of 15 (47%) patients with gliosis or other pathology. Piriform cortices did not exhibit any identifiable preoperative MRI lesions. Coexistent MRI lesions comprised extratemporal gliosis in 9/59 patients (15%) and gray‐white differentiation disorders in 5/59 patients (8%). Peri‐ and postoperative complications were present in five out of 82 patients (6%) and accounted for postoperative bleeding in one case (1%) and postoperative wound infection and meningitis in four cases (5%). Further, peri‐ and postoperative unfavorable events did not significantly impact the postoperative seizure outcome: two out of 59 (3%) patients with ILAE class 1 and three out of 23 (13%) patients with ILAE class 2–6 (*P* = 0.1) exhibited surgery‐associated complications. For further details on patient characteristics see Table 1.

### Extent of temporal piriform cortex resection predicts postoperative seizure outcome

**Figure 1:**
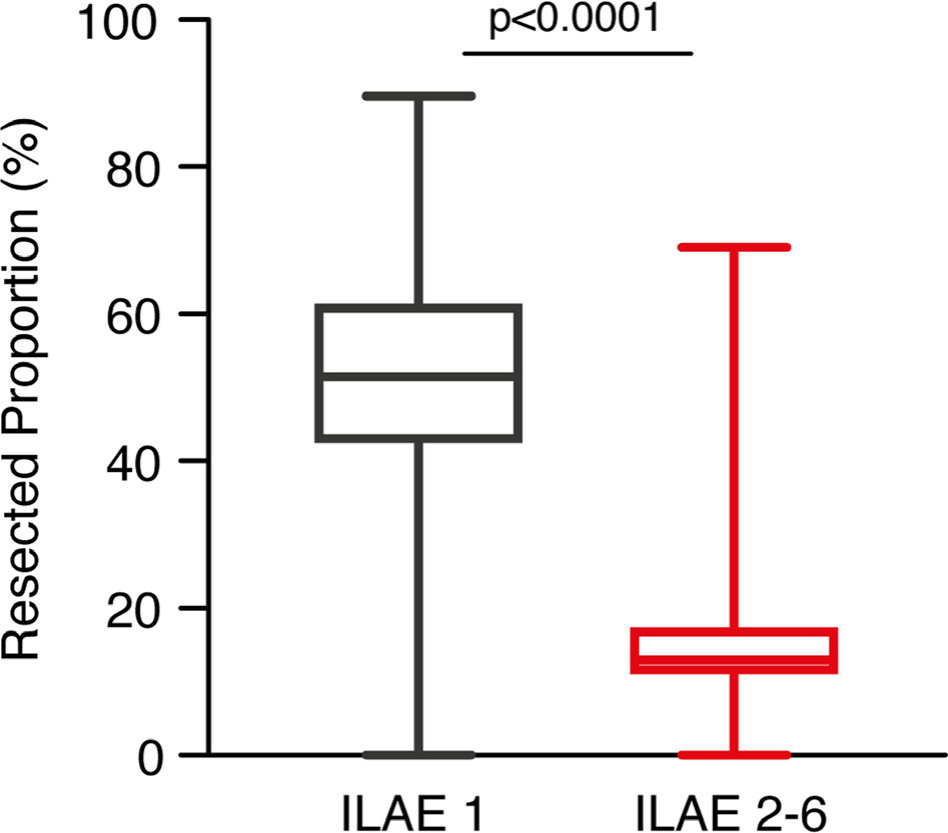
Box‐Whisker Plots illustrate seizure outcome dependent on the proportion of temporal piriform cortex resection. ILAE, International League Against Epilepsy.

While volumetric analysis of pre‐ and postoperative target volumes did not yield significant differences in the resected proportions of hippocampus and amygdala for the ILAE class 1 and ILAE class 2–6 groups (hippocampus: 81% (IQR 73–88) for favorable versus (vs.) 80% (74–92) for unfavorable seizure outcome (*P* = 0.7); amygdala: 100% (100–100) vs. 100% (100–100) (*P* = 0.6)), patients with postoperative seizure freedom revealed a profound reduction in residual piriform cortex volumes. Thereby, patients with favorable seizure outcome exhibited a median resected proportion of 51% (42–61) compared to 13% (11–18) for patients with postoperative persisting or deteriorating seizures (*P* = 0.0001) (Fig. 1, Table 2).

**Table 2:** Extent of temporal piriform cortex resection predicts postoperative seizure outcome.

	Resected proportion^1^ (median (IQR))		
	ILAE class 1 (*n* = 59)	ILAE class 2–6 (*n* = 23)	*P* Value
Piriform cortex	51 (42–61)	13 (11–18)	0.0001
Hippocampus	81 (73–88)	80 (74–92)	0.7
Amygdala	100 (100–100)	100 (100–100)	0.6

ILAE, International League Against Epilepsy; IQR, interquartile range.

^1^ Values indicated in %.

**Figure 2:**
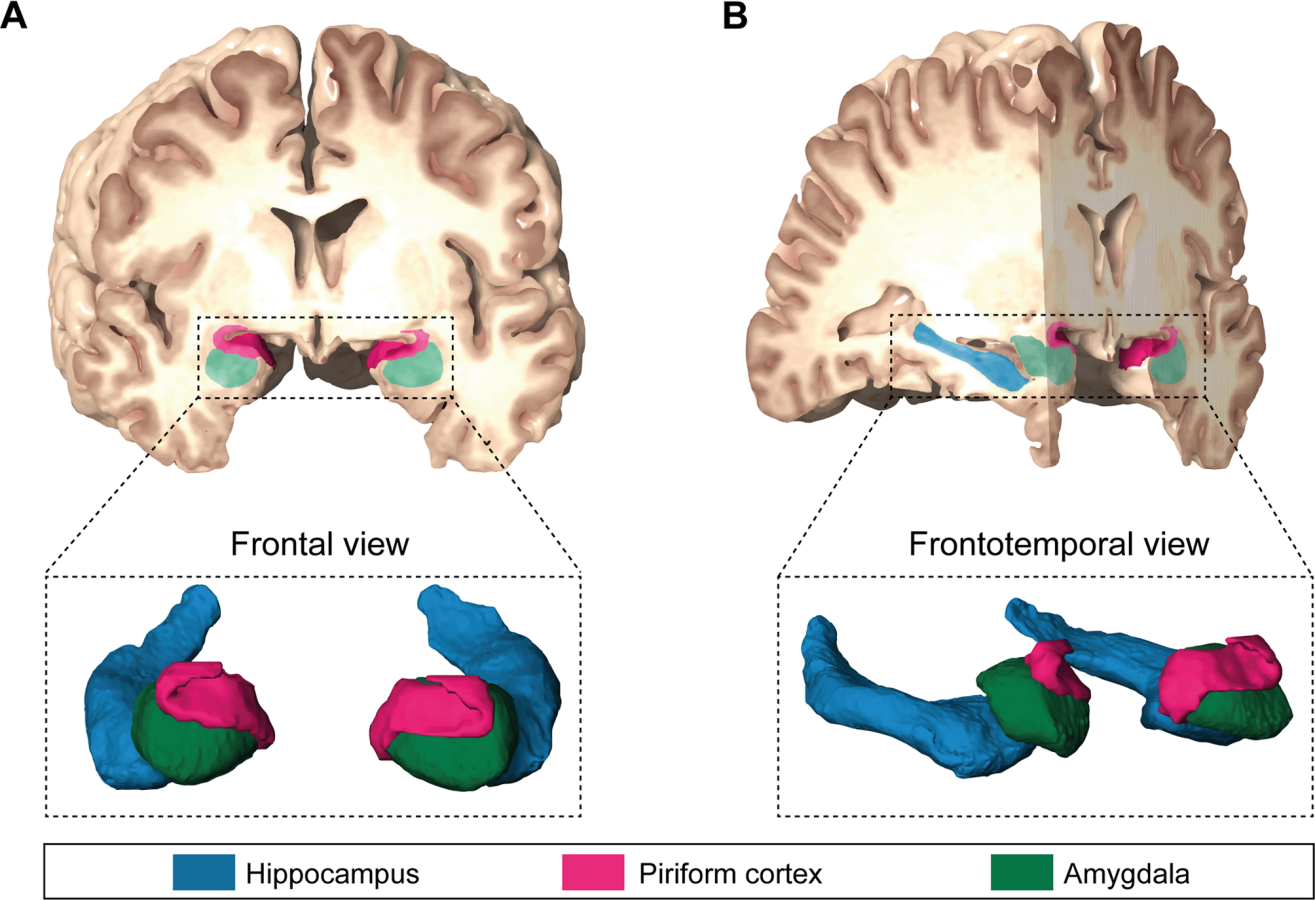
Illustration of anatomical topography of mesiotemporal target structures in tsSAHE. tsSAHE, transsylvian selective amygdalo‐hippocampectomy.

**Figure 3:**
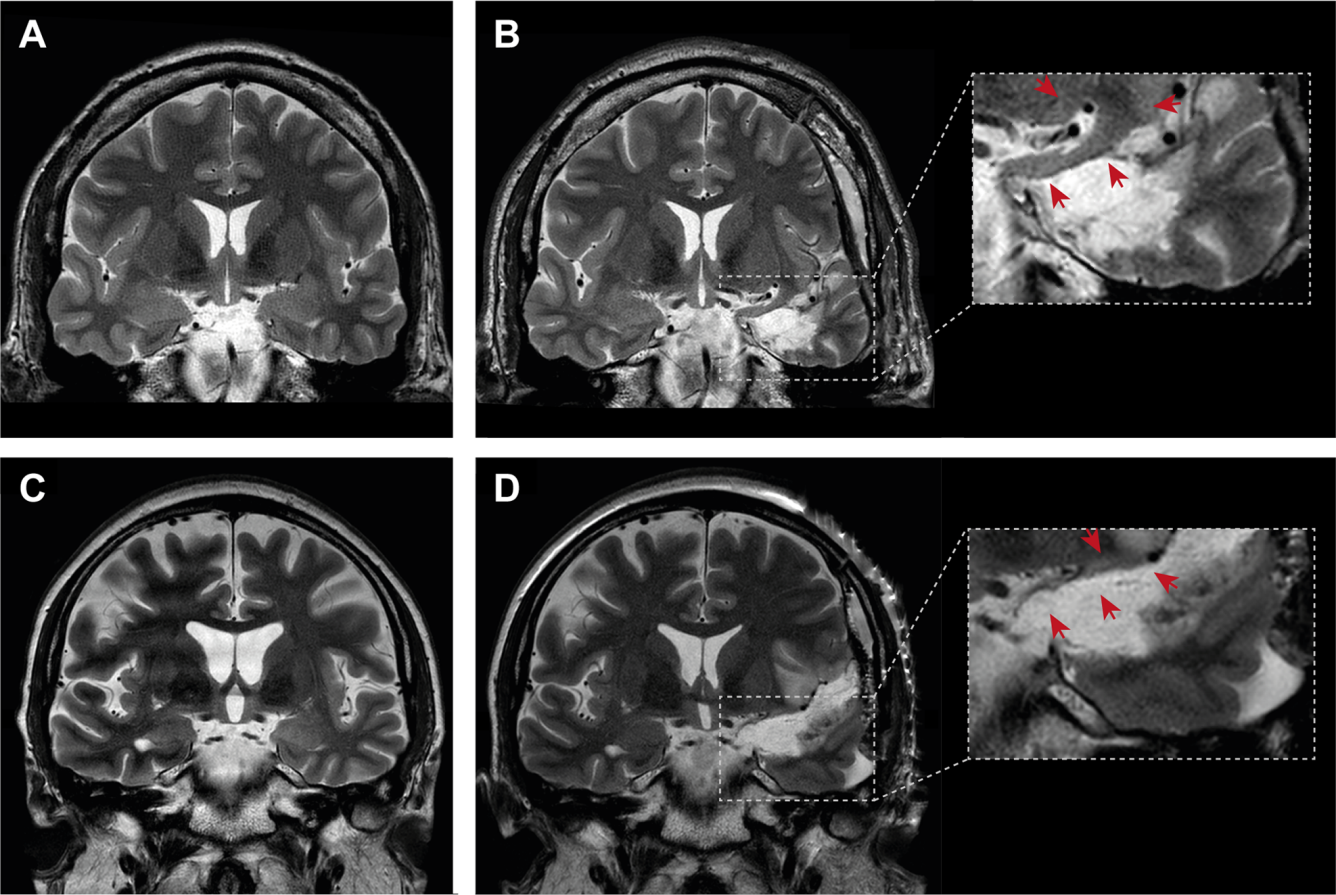
Representative coronal T2‐weighted MRI of tsSAHE with differing extent of piriform cortex resection. Pre‐(A) and postoperative (B) images show profound residual volume of piriform cortex compared to a high extent of piriform cortex resection for respective images (C) and (D). Red arrows in the enlarged sections point at postoperative residual piriform cortices.

Figure 2 illustrates the anatomical topography of hippocampus, amygdala and piriform cortex as target volumes in tsSAHE surgery. Examples of differing extents of piriform cortex resection are given in Figure 3. Preoperative volumes of abovementioned tsSAHE target structures did not significantly differ between the groups of favorable and unfavorable seizure outcome (Table 3). The volumetric analysis of tsSAHE target volumes according to affected side by the mTLE is shown in Tables S1 and S2. Additionally, we analyzed the impact of the extent of piriform cortex resection in patients with histological evidence of hippocampal sclerosis and in those without this pathology. Thereby, we did not find any significant correlation (Table 4). In order to check for a potential influence of hippocampal sclerosis and the extent of piriform cortex resection as variables both of which were significantly associated with postoperative seizure outcome in univariate analysis, a two‐level logistic regression analysis including an interaction term was performed. Thereby, we could not find any evidence for potential interactions between these two variables (*P* = 0.09).

**Table 3:** Preoperative volumetric analysis of tsSAHE target structures according to seizure outcome.

	Volumes^1^ (median (IQR))		
	ILAE class 1 (*n* = 59)	ILAE class 2–6 (*n* = 23)	*P* Value
Piriform cortex	0.39 (0.31–0.47)	0.40 (0.32–0.51)	0.7
Hippocampus	1.81 (1.54–2.36)	1.99 (1.71–2.50)	0.2
Amygdala	1.05 (0.88–1.24)	1.12 (0.93–1.41)	0.2

ILAE, International League Against Epilepsy; SD, standard deviation; tsSAHE, transsylvian selective amygdalo‐hippocampectomy.

^1^ Values indicated in ml.

**Table 4:** Extent of piriform cortex resection dependent on postoperative histological analysis.

	Proportion of piriform cortex resection^1^ (median (IQR))		
	ILAE class 1 (*n* = 59)	ILAE class 2–6 (*n* = 23)	*P* Value
Hippocampal sclerosis	50 (42–61)	13 (12–18)	0.0002
Hippocampal gliosis	62 (43–85)	13 (7–20)	0.0012

ILAE, International League Against Epilepsy; IQR, interquartile range; tsSAHE, transsylvian selective amygdalo‐hippocampectomy.

^1^ Values indicated in %.

**Figure 4:**
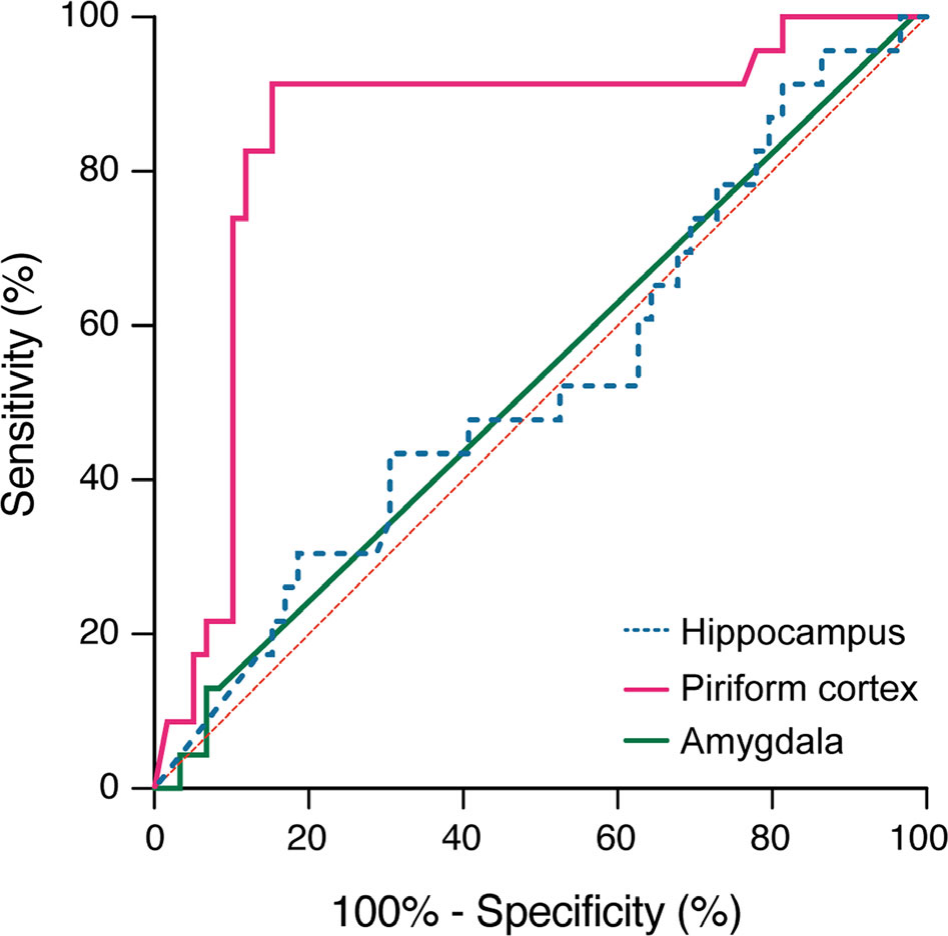
Illustration of the association between piriform cortex, amygdala and hippocampus as target structures and seizure outcome in tsSAHE. Receiver operating characteristic curves (ROC) reveals the piriform cortex as the only target volume in tsSAHE to significantly discriminate between favorable and unfavorable seizure outcome. tsSAHE, transsylvian selective amygdalo‐hippocampectomy.

The ROC analysis revealed that 50 out of 59 patients (85%) with postoperative seizure freedom (ILAE class 1) had undergone resection of more than 26.4% of preoperative temporal piriform cortex volumes. In comparison, 21 out of 23 patients (91%) with postoperative persistent or deteriorated seizures (ILAE class 2–6) exhibited resection of less than 26.4% of preoperative temporal piriform cortex volumes (Fig. 4, Table 5). As shown in Table 6, the extent of piriform cortex resection did not correlate with the new onset of neurological deficits including new visual field impairment.

**Table 5:** Seizure outcome dependent on the proportion of temporal piriform cortex resection.

	Number of patients		
	ILAE class 1	ILAE class 2–6	Total
EOR < 26%	9	21	30
EOR ≥ 26%	50	2	52
Total	59	23	82

EOR, extent of resection; ILAE, International League Against Epilepsy.

*P* < 0.0001.

**Table 6:** New postoperative neurological deficits dependent on the extent of piriform cortex resection^1^.

	EOR < 26% (*n* = 30)	EOR ≥ 26% (*n* = 52)	*P* Value
New transient motor deficit	0 (0)	1 (2)^2^	1.0
New transient aphasia	1 (3)	0 (0)	0.4
New visual deficit	17 (61)	33 (63)	0.6
quadrantanopsia	13 (43)	27 (52)	0.5
homonymous hemianopsia	4 (13)	6 (12)	1.0

EOR, extent of resection.

^1^ Values represent number of patients unless otherwise indicated (%).

^2^ transient hemiparesis.

### Neurocognitive outcome

**Figure 5:**
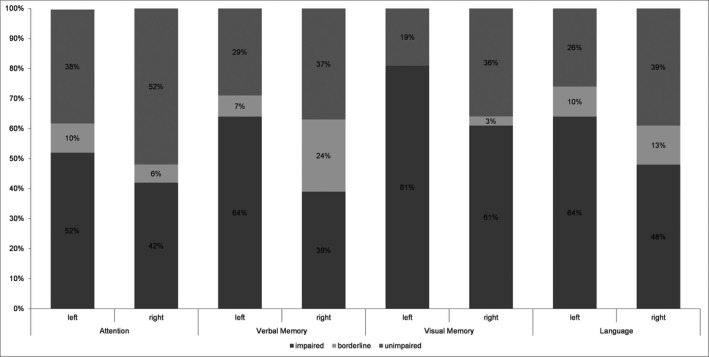
Histogram demonstrates the results of the preoperative cognitive performance. The results from each cognitive domain summarized and classified into a five‐point scale ranging from severely impaired to above average. The values represent cumulative percentage of performance categories in each tested cognitive domain according to the side of the TLE.

**Figure 6:**
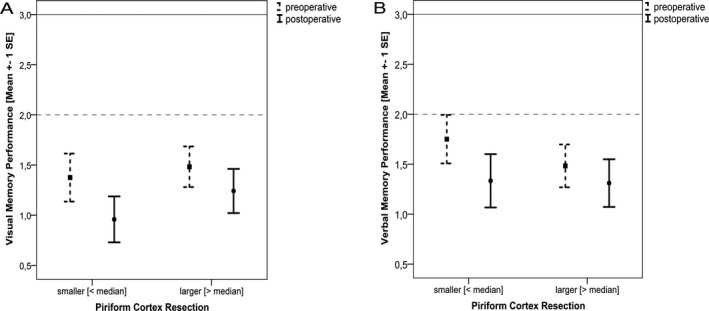
Performance in visual (A) and verbal memory (B) before and after surgery according to the extent of piriform cortex resection.

At baseline, cognitive impairments affected the majority of patients undergoing tsSAHE. Visual memory was most frequently impaired in 70% followed by verbal memory, language and attention in about 50% of the patients (Fig. 5). Postoperative assessments revealed that performance in verbal memory tasks dropped in 60% after left tsSAHE and in 27% after right tsSAHE (*X* ^2^(2) = 6.87, *P* = 0.032). Visual memory deteriorated in 33% after TLS regardless of side. In contrast, attention improved in 33%, language remained stable in 60% of the patients. Significant changes in language were found for phonemic fluency (*F*(1,39) = 7.43, *P* < 0.05, eta^2^ = 0.01) and confrontation naming (*F*(1,55) = 9.55, *P* < 0.05, eta^2^ = 0.15). Confrontation naming improved after right tsSAHE and deteriorated after left tsSAHE. Phonemic fluency improved from pre‐ to postoperative assessment. The pre‐ and postoperative memory profile for patients with a smaller resection extent (less than median) and for patients with a larger resection extent (more than median) is displayed in Figure 6.

According to regression analyses, baseline performance, surgical side, seizure outcome and extent of piriform cortex resection have proven to be good predictors of postoperative cognitive outcome explaining between 29% (attention) and 56% (language) of the variance (Table 7). Baseline performance is the best predictor for attention, memory and language. In addition, side of surgery also predicted the outcome of verbal memory. According to the β weights the extent of piriform cortex resection contributed little to the regression models.

**Table 7:** Results of regression analysis for prediction of postoperative neurocognitive outcome.

	*R* ^2^_adj._	*F*	*P*	Variable	*β*	*t*	*P*
Attention	0.29	7.07	<0.001	Baseline	0.50	4.39	<0.001
				Seizure freedom	0.11	0.29	0.77
				Surgical side	0.04	0.15	0.88
				Piriform cortex	0.01	1.94	0.06
Verbal memory	0.34	9.16	<0.001	Baseline	0.54	4.76	<0.001
				Surgical side	0.61	2.30	<0.05
				Seizure Freedom	−0.57	−1.49	0.14
				Piriform cortex	−0.01	−1.55	0.13
Visual Memory	0.51	14.85	<0.001	Baseline	0.73	7.57	<0.001
				Surgical side	−0.09	−0.43	0.67
				Seizure Freedom	0.01	0.02	0.98
				Piriform cortex	0.00	0.86	0.39
Language	0.56	17.83	<0.001	Baseline	0.60	7.10	<0.001
				Surgical side	0.33	1.96	0.06
				Seizure Freedom	−0.24	−1.04	0.30
				Piriform cortex	0.00	0.47	0.64

## Discussion

In this study, we presented results from one of the largest cohorts comparing preoperative and postoperative volumetric data on MRI exclusively in candidates suffering from mTLE and surgically treated using tsSAHE.^32^ We showed that extended resection of piriform cortex profoundly predicts seizure freedom following tsSAHE in patients with mTLE.

In 1990 Siegel et al. provided the first study on 30 patients with TLE, who had undergone tsSAHE with the focus on the relationship between seizure outcome and volumetrically estimated amount of removal within temporomesial structures.^33^ They found that smaller resection in the cranio‐caudal axis was associated with a poorer seizure outcome. Furthermore, the authors concluded that incomplete resection of the parahippocampal gyrus and the subiculum results in less favorable seizure outcome.

Recently published data by Galovic et al. comparing the association between volumetrically calculated extent of resection in the course of ATL and seizure outcome in individuals suffering from TLE^14^ suggest that seizure freedom was achieved in 60% of patients if at least 50% of piriform cortex had been resected. In contrast to the findings by Siegel et al., the analysis performed by Galovic et al. revealed no significant difference in the amount of resection of entorhinal cortex between seizure free patients and patients with continued seizures after ATL. The results of our series are in line with the findings made by Galovic and coworkers. However, in contrast with these findings, in the current series a removal of at least 26.4% of the temporal part of piriform cortex was required to achieve seizure freedom in 96% of patients following tsSAHE.

In their study, Galovic and coworkers used outlining methods for manual segmentation largely based on the publication reported by Conçalves Pereira et al. The authors reported, that particularly in the frontal lobe, the outlining of borders of piriform cortex could be difficult. Therefore, to obtain more reliable estimates of piriform cortex volumes, they focused on the temporal extention of the piriform cortex. It is important to recognize, that in the study reported by Conçalves Pereira et al., the MR images were acquired on 1.5 T MR scanner with a slice thickness of 1.5–2.0 mm. In contrast, we performed entire pre‐ and postoperative MR scans on a 3.0 T MR scanner with a slice thickness of 1.0 mm. A similar scan protocol was applied in the study by Galovic et al. According to the work reported by Vaughan and Jackson, in our study we extended the outlining of the frontal part of the piriform cortex from the endorhinal sulcus to olfactorial tubercle limiting it by the lateral olfactorial tract. In contrast to our study, Galovic et al. included only 50–75% of this distance in the volumetric analysis. In light of this aspect, the segmentation and volumetry of the frontal part of the piriform cortex was performed slightly more extensively in our study, compared to the method used by Galovic et al.

The outlining of the temporal part of piriform cortex was performed in the same fashion as reported by Galovic et al. Of note is that the frontal part of the piriform cortex was not included for volumetric analysis in the present series. This difference should be taken into account, when interpreting both these studies in regard of the required proportion of piriform cortex resection. However, the results of our study strongly support the evidence, that a more extensive resection of the temporal part of the piriform cortex is associated with a significantly higher chance to become seizure free after tsSAHE in mTLE.

Additionally, the abovementioned discrepancy in required amount of piriform cortex resection may be caused by the fact that the population in our series consists of candidates suffering from mTLE who represent a more homogeneous group of patients with a highly assumed seizure focus within the mesiotemporal structures. Therefore, extended resection of piriform cortex during tsSAHE in patients with mTLE might be more successful in removing the seizure foci compared to piriform cortex resection during ATL in patients with TLE.

Interestingly, a recent study of Wu et al. on laser interstitial thermal therapy (LITT) as a minimally invasive treatment for mesial temporal lobe epilepsy yielded superior seizure outcomes for ablation of more mesial and anterior located target structures than in the case of dorso‐lateral tracts within the hippocampal body.^34^ Notwithstanding reported data do not allow for distinct topographical analysis of piriform cortex volumes and therefore might partly explain worse overall seizure freedom rates compared to our series, these results may support the findings of strictly mesiotemporal located target volumes to significantly entail postoperative superior favorable seizure outcome rates.

In regard to neuropsychological outcome, the results in the current series are in line with previously reported studies.^19^, ^35^ Resection of piriform cortex was safe and there was no impact on neurocognitive performance in regard of extent of resection. Although the role of piriform cortex in initiation and propagation of seizures is well described in animal models, there is little evidence regarding the exact function of piriform cortex in humans.^36^, ^37^, ^38^ Therefore, further research is required to correlate the extent of resection with both seizure as well as neurocognitive outcome.

Of note is that the resection of piriform cortex using the transsylvian approach for SAHE is more challenging for the surgeon due to several aspects. One of the main limitations is a narrow operative space and restricted visualization of the temporal part of the piriform cortex. There is often a need for additional dissection of the brain tissue or even retraction in order to attempt a better visualization of the operating field. These maneuvers may be risky as parts of basal ganglia, M_1_ segment of the middle cerebral artery and other vessels traversing the anterior perforated substance could be affected. Despite this potential risk, our data strongly indicate that an effort to access and remove the temporal part of the piriform cortex should be made by the neurosurgeon during tsSAHE.

With regard to an extension of piriform cortex resection to significantly improve favorable seizure outcome, this study supports the hypothesis that the piriform cortex may profoundly be involved in the genesis of seizures in the temporal lobe. In addition to the evidence that the piriform cortex is a part of an epileptogenic network in rodent models,^39^, ^40^, ^41^, ^42^, ^43^, ^44^ there are several studies that provide evidence that piriform cortex might also be involved in the genesis and spreading of epileptic seizures in humans.^24^, ^45^, ^46^ However, when analyzing the reasons associated with failure of epilepsy surgery in mTLE, the role of piriform cortex and its extent of resection during the surgical procedure were underestimated in the literature.

One of the strengths of the present series is the homogeneous study population consisting of candidates with mTLE. Another strength is that the surgical procedure (tsSAHE) was performed in a highly standardized fashion in all patients. The imaging used for volumetric analysis was obtained from the same MRI scanner according to the standardized scanning protocol in all individuals. Despite the retrospective nature of data analysis, data acquisition was prospective. Patients were not randomized, but treated according to the decision of the interdisciplinary epilepsy surgery conference. Beyond doubt this study has several limitations. Due to its retrospective design, our study suffers from the risk of bias inherent to retrospective cohort analysis. Additionally, the present data represent a single‐center experience. However, the implementation of a standardized neurosurgical approach and strict definition of inclusion criteria and variables analyzed in the current series might mitigate some of the shortcomings of a retrospective study design.

This study provides strong evidence for temporal piriform cortex as a novel key target structure in tsSAHE surgery. With regard to a profound increase in the rate of postoperative seizure freedom following extended piriform cortex resection, the authors suggest a renewed and enhanced surgery regime. The resection strategy during tsSAHE should take into account the residual temporal piriform cortex volume as a pivotal predictor for postoperative seizure outcome in mTLE.

## Author Contributions

VB and MS conceived the study, statistical analysis, and interpretation of the data. VB and MS contributed equally to this work by designing and writing the draft of the manuscript. HV supervised the whole process of analysis and writing of the manuscript. All co‐authors made substantial contribution to the conception of the study, the treatment and recruitment of the patients and data collection. EG and PS revised the manuscript critically. All co‐authors approved the final version. RF supervised the statistical analysis and its interpretation. A‐LP and MS performed volumetric analysis after training and under continuous supervision provided by VCK and LS. The neuropsychological assessment was performed and analyzed by JT and CH. The collection of patient data was performed by GA and II. CEE and RS are responsible for the presurgical evaluation. The surgical procedures were performed by HV, VB and MH.

## Conflicts of Interest

The authors report no disclosures relevant to the manuscript.

## Supporting information


**Table S1.** Table demonstrates the results of the volumetric analysis of tsSAHE target volumes in left‐sided mTLE.

**Table S2.** Table demonstrates the results of the volumetric analysis of tsSAHE target volumes in right‐sided mTLE.
