# Supplementary material for: Resection of piriform cortex predicts seizure freedom in temporal lobe epilepsy
Source: Ann Clin Transl Neurol. 2020 Dec 2;8(1):177–89. doi: 10.1002/acn3.51263 (PMC7818082; doi:10.1002/acn3.51263)
Supplement: Supplementary file 1 — Table S1. Table demonstrates the results of the volumetric analysis of tsSAHE target volumes in left‐sided mTLE. [file ACN3-8-177-s001.docx]

**Supplementary Table S1: Volumetric tsSAHE target structure ratios in left-sided mTLE**

|  | **Volumes *****  **(median (IQR))** | | |
| --- | --- | --- | --- |
|  | **ILAE class 1**  **(n=30)** | **ILAE class 2-6**  **(n=12)** | **p-value** |
| Piriform cortex | 50 (37-66) | 15 (12-20) | 0.0007 |
| Hippocampus | 78 (70-88) | 77 (74-97) | 0.5 |
| Amygdala | 100 (100-100) | 100 (100-100) | 0.6 |

******* Values indicated in ml.

ILAE, International League Against Epilepsy; mTLE, mesial temporal lobe epilepsy; SD, standard deviation; tsSAHE, transsylvian selective amygdalo-hippocampectomy
