# Supplementary material for: Resection of piriform cortex predicts seizure freedom in temporal lobe epilepsy
Source: Ann Clin Transl Neurol. 2020 Dec 2;8(1):177–89. doi: 10.1002/acn3.51263 (PMC7818082; doi:10.1002/acn3.51263)
Supplement: Supplementary file 2 — Table S2. Table demonstrates the results of the volumetric analysis of tsSAHE target volumes in right‐sided mTLE. [file ACN3-8-177-s002.docx]

**Supplementary Table S2: Volumetric tsSAHE target structure ratios in right-sided mTLE**

|  | **Proportions of piriform cortex resection *****  **(median (IQR))** | | |
| --- | --- | --- | --- |
|  | **ILAE class 1**  **(n=29)** | **ILAE class 2-6**  **(n=11)** | **p-value** |
| Piriform cortex | 51 (43-61) | 12 (7-15) | 0.0002 |
| Hippocampus | 82 (76-89) | 84 (70-92) | 1.0 |
| Amygdala | 100 (100-100) | 100 (100-100) | 0.9 |

******* Values indicated in %.

ILAE, International League Against Epilepsy; IQR, interquartile range; mTLE, mesial temporal lobe epilepsy; tsSAHE, transsylvian selective amygdalo-hippocampectomy;
